# Inflammation Causes Mood Changes Through Alterations in Subgenual Cingulate Activity and Mesolimbic Connectivity

**DOI:** 10.1016/j.biopsych.2009.03.015

**Published:** 2009-09-01

**Authors:** Neil A. Harrison, Lena Brydon, Cicely Walker, Marcus A. Gray, Andrew Steptoe, Hugo D. Critchley

**Affiliations:** a Wellcome Trust, Centre for Neuroimaging, London, United Kingdom; b Institute of Cognitive Neuroscience, London, United Kingdom; c Department of Epidemiology and Public Health, London, United Kingdom; d Brighton and Sussex Medical School, University of Sussex Campus, Falmer, Brighton, United Kingdom

**Keywords:** Cytokines, depression, fMRI, mood, peripheral inflammation, subgenual cingulate

## Abstract

**Background:**

Inflammatory cytokines are implicated in the pathophysiology of depression. In rodents, systemically administered inflammatory cytokines induce depression-like behavior. Similarly in humans, therapeutic interferon-α induces clinical depression in a third of patients. Conversely, patients with depression also show elevated pro-inflammatory cytokines.

**Objectives:**

To determine the neural mechanisms underlying inflammation-associated mood change and modulatory effects on circuits involved in mood homeostasis and affective processing.

**Methods:**

In a double-blind, randomized crossover study, 16 healthy male volunteers received typhoid vaccination or saline (placebo) injection in two experimental sessions. Mood questionnaires were completed at baseline and at 2 and 3 hours. Two hours after injection, participants performed an implicit emotional face perception task during functional magnetic resonance imaging. Analyses focused on neurobiological correlates of inflammation-associated mood change and affective processing within regions responsive to emotional expressions and implicated in the etiology of depression.

**Results:**

Typhoid but not placebo injection produced an inflammatory response indexed by increased circulating interleukin-6 and significant mood reduction at 3 hours. Inflammation-associated mood deterioration correlated with enhanced activity within subgenual anterior cingulate cortex (sACC) (a region implicated in the etiology of depression) during emotional face processing. Furthermore, inflammation-associated mood change reduced connectivity of sACC to amygdala, medial prefrontal cortex, nucleus accumbens, and superior temporal sulcus, which was modulated by peripheral interleukin-6.

**Conclusions:**

Inflammation-associated mood deterioration is reflected in changes in sACC activity and functional connectivity during evoked responses to emotional stimuli. Peripheral cytokines modulate this mood-dependent sACC connectivity, suggesting a common pathophysiological basis for major depressive disorder and sickness-associated mood change and depression.

Clinical and animal studies implicate systemic inflammation in the pathogenesis of depression (1). In healthy mammals, systemic infection triggers profound behavioral changes, including cognitive and mood symptoms (e.g., memory impairment, anhedonia, anxiety, depression), change in motivation (anorexia, adipsia), and neurovegetative symptoms (sleep disturbance, psychomotor slowing) (2–4) known as sickness behaviors. Animal and human studies suggest that inflammatory cytokines play a central role in mediating these sickness-related behaviors by communicating peripheral inflammation to the brain. These cytokine-induced sickness behaviors show a striking similarity to symptoms of major depression (5) and might account for the high incidence of depression in medically ill patients, particularly those with inflammation or immune activation secondary to infection, autoimmunity, tissue damage, or malignancy.

In rodents, systemic administration of interleukin (IL)-1β or bacterial lipopolysaccharide (LPS), a potent stimulant of cytokine release, can rapidly elicit a depression-like syndrome characterized by a reduction in positively motivated approach behaviors such as exploration, social interaction, and in operant behaviors for food reward (6–9). Similarly experimental induction of inflammation in healthy human subjects with either LPS (4) or typhoid vaccination (10,11) acutely induces symptoms of fatigue, psychomotor slowing, mild cognitive confusion, memory impairment, anxiety, and deterioration in mood that mirror features of depression. Furthermore, patients receiving more prolonged or repeated therapeutic administration of interferon (IFN)-α show that inflammatory cytokines induce true major depressive episodes in up to 50% of individuals (12). Moreover, in patients with major depressive disorder (MDD), the presence of high levels of pro-inflammatory cytokines (in particular IL-6) (13) and acute phase proteins (14) suggest that inflammatory mediators might contribute to the pathophysiology of depression even in the absence of medical illness.

Depression is increasingly recognized to be a multi-componential disorder involving motivational change; cognitive, attention, memory, and mood disturbance; and biological features such as disturbed appetite, sleep, and sexual dysfunction. It is therefore interesting that separate features of clinical depression induced by repeated administration of IFN-α over many weeks seem to develop with characteristic time-courses (12,15). “Neurovegetative” symptoms such as fatigue, psychomotor slowing, anorexia, and impaired sleep develop early, typically within 2 weeks of initiation of IFN-α therapy (12), whereas subjective reports of depressed mood, anhedonia, anxiety, irritability, memory, and attentional disturbance assessed with clinical depression scales usually develop later, between the first and third months of IFN-α therapy (12). The neurobiological basis for differential evolution of individual features of inflammation-associated clinical depression is currently uncertain; however, the ability of acute inflammation to rapidly induce multiple depression-like symptoms suggests that the immune system can rapidly modulate neuronal circuits central to the organization and reorganization of complex motivational behavior that might lead to the establishment of mood disorder.

In a separate report published in this journal, we use functional magnetic resonance imaging (fMRI) to show that *Salmonella typhi* vaccine-induced inflammation modulates activity within the hierarchy of brain regions representing internal bodily state (16). These observations in human participants are consistent with rodent studies that suggest cytokines act on autonomic afferent nerves to mediate motivational reorientation during the early phase of inflammation (17,18). Furthermore, this and another recent study showed that fatigue and psychomotor slowing were associated with corresponding changes in activity within insula (16) and substantia nigra (19).

The current study was designed to investigate the neurobiological mechanisms through which inflammation induces an acute deterioration in mood, via effects on emotional processing. In particular, we wished to investigate whether inflammation-associated deterioration in mood recruited circuits implicated in the pathophysiology of depression. As a model of emotional processing we chose a variation of a face perception task that is known to activate the amygdala, superior temporal sulci (STS), and fusiform cortex in a mood-dependent manner (20).

## Methods and Materials

Sixteen healthy male students, mean age (± SD) 24.9 (± 4.8) years, were recruited from University College London (UCL) campus advertisements. Ten were Caucasian, 3 Indian-Asian, 2 Chinese-Asian, and 1 Latin-American. Volunteers were reviewed by a psychiatrist (NAH) and screened for a history of any relevant physical or psychiatric illness. One participant had a history of hay fever, and another had shellfish allergy. Four participants rated their general health as excellent, 7 very good, and 5 good. No participant rated their general health as poor or fair. All were medication free, with no nonsteroidal or steroidal inflammatory drug use in the preceding 2 weeks, and were nonsmokers. Volunteers who had received typhoid vaccine within 3 years or other vaccine within 6 months were excluded. Participants were advised to not consume caffeinated beverages or alcohol, avoid high-fat meals, and refrain from excessive exercise for 12 hours before testing. They were asked not to take aspirin, ibuprofen, or antibiotics for 14 days before testing. After complete description of the study to the subjects, written informed consent was obtained. Procedures were approved by the joint UCL/UCL Hospital Ethics Committee. Findings in the same group of participants are also reported elsewhere (19,16).

**Figure 1:**
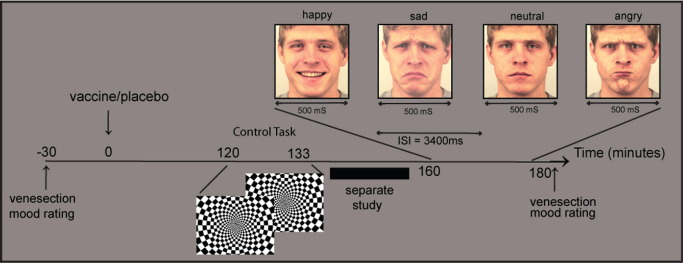
Study timeline. Participants completed mood rating questionnaires (Profile of Mood States [POMS]) and underwent venesection, then randomly received *Salmonella typhi* capsular polysaccharide vaccination (Typhim Vi, Aventis Pasteur MSD, Berkshire, United Kingdom) or normal saline placebo injection on two separate occasions 1 week apart. Two hours after injection, participants completed a flashing checkerboard control task, then an implicit facial emotion recognition task during functional magnetic resonance imaging. The POMS and venesection were repeated at 3 hours. Facial expressions reproduced with permission from the Section of Psychology, Department of Clinical Neuroscience, Karolinska Institutet, Stockholm, Sweden (23).

We adopted a randomized, double-blind, cross-over repeated measures design in which all participants underwent imaging in two separate sessions, an average of 7 days apart as reported previously (16). In the first session participants were randomly assigned to one of two experimental conditions (typhoid vaccine or placebo). Baseline blood sample was taken; then injections of .025 mg of *Salmonella typhi* capsular polysaccharide vaccine (Typhim Vi, Aventis Pasteur MSD, Berkshire, United Kingdom) or .5 mL of normal saline placebo were administered intramuscularly into the deltoid muscle. An fMRI was performed 2 hours after injection in a 60-min session. During each session, participants performed three tasks. This article focuses on data acquired during an implicit facial-affect processing task. Immediately after scanning, a second blood sample was taken (3 hours after injection) for cytokine measurement. Body temperature was assessed at baseline and at 2 and 3 hours with a sublingual digital thermometer. See Figure 1 for study timeline. The second session was identical except that participants received the other injection (i.e., typhoid vaccination if they previously received saline and vice versa).

Mood and other psychological symptoms were assessed with a modified version of the Profile of Mood States (POMS) (21). This consisted of 36 items, each of which was rated on a five-point scale (0 = not at all to 4 = extremely). Six items were taken from the vigor, tension-anxiety, depression-dejection, and confusion scales of the POMS and five from the fatigue scale. In addition, there were four symptom (feverish, aching joints, nauseated, and headache) and three filler items. Participants were asked to rate how they felt at that moment. Scores for the five POMS subscales were computed by summing ratings on individual items. Total mood scores were derived by the standard method detailed in the POMS rating manual of subtracting ratings on the negative scales (tension-anxiety, depression-dejection, confusion, and fatigue) from the vigor scores (21). Of note, this method produces a composite total mood score that is sensitive to changes in cognitive-mood and neurovegetative contributions to mood.

We adopted a model of mild experimental inflammation with standard typhoid (*Salmonella typhi*) vaccination that has previously been shown to induce both a low-grade toll-like-receptor-4 mediated inflammatory-cytokine response (associated with an approximate doubling of peripheral IL-6 levels peaking between 2 and 3 hours) (22) and a transient negative total mood (peaking 1.5–3 hours after injection) (10,11). Injection of .025 mg *Salmonella typhi* vaccine (Typhim Vi, Aventis Pasteur MSD) or .9% sodium chloride placebo in identical 2-mL syringes was administered intramuscularly into the deltoid muscle by a qualified doctor (NAH). There were no complications of either injection.

Separate venepunctures were performed at baseline and 3 hours after injection for vaccine and placebo conditions. Blood (10 mL) was drawn into Vacutainer tubes (Becton Dickinson and Company, Franklin Lakes, New Jersey) containing ethylenediaminetetraacetic acid (EDTA) anticoagulant, centrifuged immediately at 1250 *g* for 10 min at room temperature. Plasma was removed, aliquoted, and frozen at −70°C before analysis. Plasma IL-6 and tumor necrosis factor α (TNF-α) were assessed with high-sensitivity, two-site enzyme-linked immunosorbent assays (ELISAs) (R&D Systems, Oxford, United Kingdom). The limit of detection of the IL-6 assay was .09 pg/mL, with intra- and interassay coefficients of variation (CVs) of 5.3% and 9.2%, respectively. The TNF-α assay had a detection limit of .10 pg/mL with intra- and interassay CVs of 6.9% and 8.4%, respectively. Plasma IL-1RA concentrations were determined by a commercial ELISA from R&D Systems. This assay had a limit of detection of 15 pg/mL and inter- and intra-assay CVs of <10%. Salivary cortisol was collected with cotton dental rolls at baseline and at 2 and 3 hours (Salivettes, Sarstedt, Leicester, United Kingdom) and analyzed with a time resolved immunoassay with fluorescence detection. Intra- and interassay variability were <8%.

Twenty faces (10 male) from a standardized series of facial emotional expressions (Karolinska-Directed-Emotional-Faces-Set [KDEF]) (23) were selected displaying happy, sad, angry, and neutral expressions. Each face was presented for 500 msec in random order with an intertrial interval of 3400 msec. Each identity-expression combination was presented 4 times, along with 48 baseline trials (cross hair fixation). The same stimulation set was used after both vaccine and normal saline placebo injection. Participants performed an incidental age judgment task: indicating with a right-handed response pad if older or younger than 25 years of age. An orthogonal task and short stimulus presentation were chosen to elicit incidental affective processing that previous data suggest might precede explicit processing (24).

Gradient-echo single-shot echo planar imaging was used to acquire T2*-weighted image volumes on a 1.5-T Siemens Sonata (Siemens AG Medical Solutions, Erlangen, Germany) scanner equipped with a standard head-coil. External restraint was used to minimize head movement. We acquired 284 volumes each with 44 slices (contiguous 2-mm slices with 1-mm inter-slice gap, echo-time 40 msec: spatial resolution 3 mm × 3 mm × 3 mm). Slices were tilted −30° from the intercommissural plane to reduce orbitofrontal dropout due to susceptibility artifact from frontal sinuses (25). High-resolution inversion-recovery echo planar images were also obtained to aid image registration.

The fMRI data were analyzed with SPM5 (http://www.fil.ion.ucl.ac.uk/spm). The first 5 volumes were discarded to allow for T1 equilibration. Individual scans were realigned, unwarped, normalized, and spatially smoothed with an 8-mm full-width-at-half-maximal Gaussian kernel with standard SPM methods. High-pass frequency filter (cut-off 120 sec) and corrections for auto-correlation between consecutive scans (auto-regressive [AR]1) were applied to the time series. Each event was modeled by a standard synthetic hemodynamic response function at each voxel across the whole brain. Presentations of neutral, happy, sad, and angry facial expressions were modeled as separate regressors. Null events (15% of presentations) were included to facilitate identification of hemodynamic responses to stochastically ordered stimuli.

First-level individualized design matrices were estimated in the following manner: effects of task (viewing happy, sad, angry, and neutral facial expressions) were computed on a voxel-wise basis for each participant for both vaccination and placebo conditions in the form of SPMs of discrete contrasts within the general linear model. Subsequent second-level analyses were performed on the SPM contrast images with a 4 (emotional expression) × 2 (inflammatory status) factorial design to permit formal inferences about population effects.

The main effect of viewing facial expressions (across both vaccine and placebo conditions) was calculated within a second-level analysis of variance encompassing individual contrasts for each of the emotional expressions. Functionally activated clusters in bilateral fusiform gyrus (fusiform face area [FFA]) and STS were identified at a family-wise error corrected threshold (FWE) of *p <* .05 and extracted with the image analysis package MarsBaR (26). Anatomical localizers from the same package were used for the amygdala bilaterally. Effects of inflammation on activity in each of these regions was determined by extracting the contrast estimates from the peak voxel in each region and analyzing it in a 4 (emotional expression) × 2 (inflammatory status) repeated measures analysis of variance in SPSS (SPSS, Chicago, Illinois).

We then performed a between-subject analysis to determine regions in which response to implicit observation of emotional facial expressions was modulated as a function of inflammation-associated total mood change. This whole brain regression analysis was performed with individual activation maps to facial emotional expressions (vs. implicit baseline) with inflammation-associated mood change as the between-subject dependent variable. Results are reported for the STS, FFA, and amygdala at uncorrected and stringent small volume FWE corrected thresholds.

We extended this approach to determine the effects of inflammation-associated mood change on brain regions previously implicated in the etiology of depression, targeting specific cortical (sACC, medial prefrontal, anterior cingulate) and subcortical (thalamus, nucleus accumbens) regions of interest, informed by prior research by Mayberg *et al.* (27) and others. This whole brain between-subject regression analysis (with inflammation-associated total mood change as the dependent variable) identified bilateral sACC as the region showing the strongest positive correlation with inflammation-associated deterioration in total mood in keeping with previous analyses on the neurobiological basis of depression (27) and anhedonia (28). Of note, no region outside of this predefined region of interest showed a significant correlation with inflammation-associated mood change at an uncorrected threshold of *p* < .001, 10 contiguous voxels.

We next performed an effective connectivity analysis to test for changes in interregional neural connectivity related to the psychophysiological interaction (PPI) between activity in sACC (physiological variable) and reported changes in total mood (psychological variable) (i.e., which brain regions increase or decrease their connectivity to sACC in a mood-dependent manner). Results are reported for uncorrected and stringent whole brain or region of interest FEW-corrected for multiple comparisons. Finally we used peripheral cytokine response (IL-6), as an index of peripheral inflammation, in correlational analyses to determine the influence of peripheral cytokines on emotional face processing, its interactions with inflammation-associated mood change, and mood-dependent connectivity of sACC.

## Results

Participants showed a significantly greater increase in serum IL-6 at 3 hours after typhoid vaccination compared with placebo injection (mean difference [± SE] vaccine 1.00 [± .21] pmol/L vs. placebo .27 [± .13] pmol/L) [paired *t*(15) = 2.84, *p* = .01], confirming a robust inflammatory response. Increase in serum IL-1RA and TNF-α after vaccine did not reach significance, *p* > .05 (see Figure 1 in our accompanying article [16]). No subject had previously received typhoid vaccination, so these reflect primary immune responses. Changes in salivary cortisol and core body temperature with time were not significantly different between vaccination and typhoid conditions, although both showed significant reduction with time in both conditions (see Supplement 1 in accompanying [16]). Of note, this temperature reduction likely resulted from being in a cool scanning environment for 1 hour. Mean change salivary cortisol (± SE): vaccine −2.85 (± 3.31) nmol/L, placebo −5.63 (± 1.76) nmol/L [paired *t*(15) = .71, *p* = .49]. Mean change body temperature: vaccine −.71°C (± .14°C), Placebo −.46°C (± .19°C) [paired *t*(15) = 1.13, *p* = .28].

Participants reported a significant deterioration in total mood 3 hours after vaccination [single-tailed paired *t*(15) = 1.86, *p* = .041] but not after placebo [*t*(15) = 1.43 *p* = ns]; this accords with findings in two previous studies reporting POMS total mood responses to typhoid vaccination (10,11). At baseline subjects showed a high positive vigor score coupled with low ratings on negative scales, indicating that participants were generally in positive moods. More negative total mood was associated with higher trait anxiety, as has been observed previously (10). Examination of the subscales contributing to the POMS total mood score showed that inflammation-associated change in total mood score was driven by an increase in both mood/cognitive (confusion) and neurovegetative (fatigue) symptoms. Smaller contributions to change in total mood score came from an increase in inflammation-associated tension-anxiety. Changes in depression-dejection and vigor subscales showed little contribution suggesting that inflammation-associated changes in total mood largely reflected increases in confusion, fatigue, and to a lesser extent tension-anxiety rather than depression-dejection or vigor (see Supplement 1 in [16]). Although we did not observe a significant linear correlation between total mood change and magnitude of IL-6 response, there was a trend toward a greater deterioration in mood in those individuals with the greatest IL-6 responses [*T*(15) = −1.77, *p* = .099, *r* = −.43]. There was no effect of vaccine on ratings of illness symptoms, including fever, nausea, aching joints, or headache.

Viewing all facial expressions (across placebo and vaccine conditions combined) activated the brain regions supporting emotional face perception, including face-selective fusiform cortex (29), superior temporal sulci, and bilateral amygdala (Table 1). Additional task–specific activations at the same stringent FEW-corrected threshold, *p* < .05, were seen in primary visual, sensorimotor, insula, premotor, dorsal anterior cingulate, and right dorsolateral prefrontal cortices (DLPFC).

Emotional compared with neutral expressions were associated with significantly greater activity in the left amygdala and bilateral superior temporal sulci, regions previously implicated in viewing emotionally valenced faces. There was no significant difference in activity in face-selective regions of fusiform cortex (Table 2), consistent with the incidental nature of the task. Secondary emotional enhancement of fusiform face cortex activity is largely task-dependent, with some studies reporting an effect (30) and others none (31). Inflammation had a marginally significant effect on right-sided STS responses to viewing emotional facial expressions [*F*(15,1) = 4.37, *p* = .055] with no significant influence on activity in any of the other face response regions reported in Table 1.

**Figure 2:**
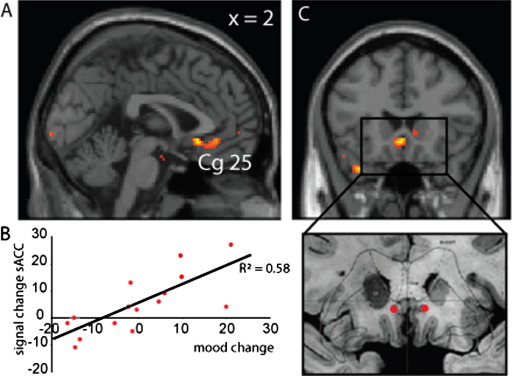
Subgenual cingulate (subgenual anterior cingulate cortex [sACC], Cg25) activity predicts inflammation-associated total mood change. **(A)** Region of subgenual cingulate (Cg25), which shows the strongest prediction of inflammation-associated deterioration in total mood. **(B)** Correlation of activity in an 8-mm diameter region of interest centered on the peak subgenual cingulate voxel (−2, 22–28) (ordinate) with inflammation-associated total mood change (abscissa). **(C)** Area of placement of deep brain stimulation electrodes for the treatment of primary depression (27) showing relative position with respect to activation shown in **A**. Reprinted from *Neuron*, volume 45, Mayberg *et al.*, “Deep brain stimulation for treatment-resistant depression,” 651–660, copyright 2005, with permission from Elsevier (27).

The significant deterioration in total mood after inflammation but not placebo at 3 hours was highly correlated with enhancement of evoked responses to emotional facial expressions within the sACC (including cytoarchitectonically defined Cg25), Montreal Neurological Institute (MNI) coordinates (−2, 22, −28) (Figures 2A and 2B, Table 3). This region is critically implicated in neurobiological mechanisms of depression and the target for successful treatment of depression with deep brain stimulation (Figure 2C). Activity within amygdala, a key region in processing emotional information from faces, was correspondingly attenuated (Table 1). The sACC activity reflected the interaction between processing of emotional facial expressions and inflammation-associated total mood change rather than as a main effect of processing emotional faces.

**Figure 3:**
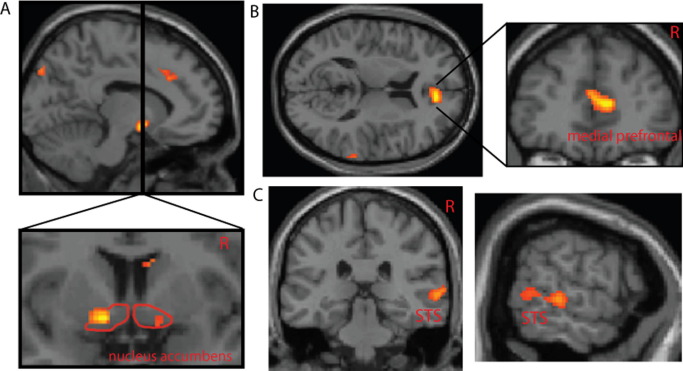
Psychophysiological interaction between inflammation-associated total mood change and subgenual cingulate (Cg25) activity. **(A)** Inflammation-associated change in total mood correlates with reduction in connectivity between subgenual cingulate and bilateral nucleus accumbens. **(B)** Region of anterior rostral medial prefrontal cortex showing reduced connectivity to subgenual cingulate with greater inflammation-induced mood change. **(C)** Inflammation-associated change in total mood correlates with reduced subgenual cingulate connectivity to superior temporal sulcus.

We extended the investigation of this interaction with a connectivity analytic approach (PPI). Specifically, we first determined brain regions where activity change to processing emotional faces correlated with sACC activity (effective connectivity), then examined how inflammation-associated total mood change modulated connectivity to sACC (Table 3). Individuals who experienced the greatest deterioration in total mood after peripheral inflammation showed a highly significant reduction in the functional relationship between sACC and activity within anterior rostral medial prefrontal cortex (arMPFC), (Brodmann area [BA]32, BA10), MNI coordinates (10, 48, 8), nucleus accumbens (Figures 3B and 3A, respectively), right amygdala, STS (Figure 3C), and FFAs. No a priori region in mood-related circuits showed increased connectivity to sACC with enhanced depression.

Finally we investigated whether peripheral inflammatory cytokine levels (IL-6) influenced sACC activity or its mood-dependent connectivity. Although circulating IL-6 level did not correlate with neural responses to emotional faces within sACC directly (or any other brain region), it did show a modulatory influence on the mood-dependent connectivity of sACC to right amygdala (*R*^2^ = .49, *p* = .004), nucleus accumbens (*R*^2^ = .33, *p* = .025), arMPFC (*R*^2^ = .37, *p* = .016), and right STS (*R*^2^ = .31, *p* = .032).

## Discussion

The results of this study illustrate that experimental inflammation induced by typhoid vaccination produces a robust increase in pro-inflammatory cytokines (IL-6) and reduction in a composite measure of mood (that includes both cognitive-mood and neurovegetative contributions) in normal healthy male volunteers at 3 hours. This was not associated with an increase in temperature or salivary cortisol or ratings of illness symptoms, including fever, nausea, and aching joints, suggesting that the reduction in total mood is likely a direct result of associated cytokine responses. As has been observed previously, greater sensitivity to inflammation-associated deterioration in this mood measure was associated with higher trait-anxiety (10). This is in keeping with the broader literature on predisposing factors for clinical depression (32) and studies of IFN-α–induced depression where personality traits predict patients most at risk of developing depression during treatment of hepatitis C or malignant melanoma (33).

Evidence from functional brain imaging, therapeutic, and lesion studies have led to a conceptualization of depression as a multidimensional systems level disorder affecting discrete but functionally integrated pathways (34,35). The sACC has become increasingly recognized as a key node in functional and anatomical models of mood regulation (36) and coordination of emotional processing. It is also strongly implicated in the pathophysiology of MDD (37). Increased sACC activity seen in depression has also been shown to reverse with successful depression treatment with a selective serotonin reuptake inhibitor (38,39), deep brain stimulation (27,40) of adjacent white matter tracts, and even placebo (39). It is therefore striking that inflammation-associated deterioration in total mood correlated with activity evoked by emotional facial expressions in sACC. These early mood-dependent changes in sACC activity occurring within 3 hours of inflammatory challenge suggest that inflammation-associated changes in this composite mood measure recruits neural circuitry similar to that implicated in primary depression. This is also supported by the responsiveness of cytokine-related mood changes to selective serotonin reuptake inhibitor treatment.

Our connectivity analysis showed that—consistent with theoretical models of mood regulation—inflammation-associated changes in total mood modulated the connectivity of sACC and nucleus accumbens with reduction in effective interconnectivity predicting greater deterioration in total mood. These findings support animal studies showing that inflammation-associated reduction in positively motivated, depression-like behavior correlates with reduced nucleus accumbens activity (8). The mood-dependent modulation of nucleus accumbens activity in our study highlights a potential mechanism for inflammation-induced modulation of hedonic tone through changes in sACC influences on reward-related brain regions and might underlie anhedonia, another core feature of depression (28).

Inflammation-associated deterioration in total mood was also associated with a marked decrease in activity within amygdala, a region central to processing emotional information from faces. Connectivity analysis also showed that inflammation-associated mood change modulated the connectivity between sACC and arMPFC, a region activated when thinking about others, and with right STS and amygdala, regions implicated in processing social/emotional information from faces. These changes might underpin the marked reduction in social behavior associated with acute sickness, possibly reflecting an internal self-reorientation of attentional focus (9) and the heterogeneity of symptoms associated with inflammation-associated mood change. Interestingly, depression is typically associated with an increase in amygdala activity (41) to emotional faces (42), suggesting that sustained mood change might modulate connectivity within this circuitry. It is noteworthy that our composite measure of mood change that was sensitive to heterogeneous mood-related symptoms also revealed modulation of activity within a network of regions connected to sACC, a region itself implicated in integrating multiple components of mood homeostasis. Future studies should focus on whether differential influences of inflammation on each of these circuits underlie differences in the temporal evolution of individual components of depression associated with repeated and prolonged cytokine administration.

In the present study, the correlation between peripheral cytokine levels (IL-6) and inflammation-associated mood deterioration did not reach significance, although there was a trend for those subjects showing the greatest IL-6 response to report a greater deterioration in total mood [*t*(15) = −1.77, *p* = .099, *r* = −.43]. Previously, Wright *et al.* (11) showed a significant correlation between IL-6 response and subjective mood deterioration with a similar effect size with the same typhoid vaccination inflammatory model and POMS total mood score. However, this study used a larger sample of 30 healthy male volunteers. The evoked inflammatory responses in our study also did not directly predict changes in the magnitude of activity within sACC, a reported observation for TNF-α responses to inflammatory challenge in subjects with asthma (43). However, IL-6 responses did influence the mood-dependent connectivity of sACC with amygdala, arMPFC, nucleus accumbens, and STS, suggesting that inflammation-associated deterioration in total mood arose though the modulation of sACC connectivity with regulatory centers for reward, emotion, and social processing. It is plausible that a more direct influence of peripheral inflammatory mediators on sACC activity might have been obscured by constitutional differences of participants, including gender and age (our current study was performed in young healthy male subjects, whereas the study population of Rosenkranz *et al.* [43] in asthma subjects was 50% female). This question will need to be addressed in future studies performed in larger and more heterogeneous populations.

Our findings provide a mechanistic neurobiological account for heterogeneous mood-related components of sickness behavior and suggest a common pathophysiological basis for MDD and sickness-associated mood change and depression. Putatively, these observations imply that the central neurobiological circuits supporting adaptive motivational reorientation during sickness might be “hijacked” maladaptively during clinical depression.

## Figures and Tables

**Table 1:** Regions Responsive to Emotional Faces

Side Region (MNI)	Coordinates (x y z)			*Z* Score	Uncorrected *p*	Whole Brain Corrected (FWE) *p*
Predicted ROI						
R fusiform gyrus	36	−62	−20	>8	<.001	<.05
L fusiform gyrus	−42	−58	−24	7.79	<.001	<.05
L STS	−54	−56	14	5.86	<.001	<.05
R STS	54	−38	8	4.99	<.001	<.05
L amygdala	Anatomical ROI			5.17	<.001	<.05
R amygdala	Anatomical ROI			4.57	<.001	<.05
Other Activated Regions						
R DLPFC	−48	44	10	7.41	<.001	<.05
L 1° Sensori-motor cortex	−44	−22	60	7.11	<.001	<.05
Bi primary visual cortex	±6	−90	8	7.01	<.001	<.05
L premotor and DAC	−4	8	56	6.86	<.001	<.05
R cingulum	16	−28	−10	5.94	<.001	<.05
R insula	38	14	−8	5.27	<.001	<.05

MNI, Montreal Neurological Institute; FWE, family wise error; ROI, region of interest; STS, superior temporal sulcus; DLPFC, dorsolateral prefrontal cortex; DAC, dorsal anterior cingulate.

**Table 2:** Modulation in Face Responsive Regions to Emotional Expression

Side Region (a) Emotion > Neutral ROI	Coordinates (x y z)	*Z* Score	K Cluster^a^	Uncorrected *p*	Small Volume Corrected *p*
L STS	(−56, −46, −4)	2.85	10	<.002	<.07
R STS	(58, −32, 0)	3.21	20	<.001	<.05
L amygdala	(−28, 2, −18)	3.50	29	<.001	<.05
R+L fusiform face area		ns		ns	ns

STS, superior temporal sulcus.

a Cluster size at uncorrected *p* value reported in the table.

**Table 3:** Regions Whose Activity and Connectivity to sACC Predicts Inflammation-Associated Mood Change

Side Region	Coordinates (x y z)	*Z* Score	*R*	Uncorrected *p*	Small Volume Corrected *p*
Activity^a^					
R+L sACC (Cg25)	(−2, 22, −8)	3.29	.55	<.001	<.05
L amygdala	(−14, −8, −28)	3.39	.57	<.001	.07
Connectivity^b^					
R+L medial frontal gyrus	(10, 48, 8)	5.21	.88	<.001	<.05^c^
L nucleus accumbens	(−10, 2, −10)	4.99	.85	<.001	<.05
R fusiform face area	(44, −48, −16)	3.15	.51	<.001	ns
R STS	(62, −20, 0)	3.61	.62	<.001	<.05
R amygdala	(26, −6, −18)	3.04	.54	<.001	<.05

*R*^2^ values correspond to peak voxel. sACC, subgenual anterior cingulate cortex; STS, superior temporal sulcus.

a Inflammation-induced mood change.

b Functional connectivity to subgenual cingulate (Cg25).

c Whole brain family wise error-corrected *p* value.
